# Psychosocial mediators of perceived stigma and suicidal ideation among transgender women

**DOI:** 10.1186/s12889-020-8177-z

**Published:** 2020-01-29

**Authors:** Krishna Kiran Kota, Laura F. Salazar, Rachel E. Culbreth, Richard A. Crosby, Jamal Jones

**Affiliations:** 1 Department of Health Policy & Behavioral Science, School of Public Health, Georgia State University, PO Box 3984, Atlanta, GA 30302-3984 USA; 2 Department of Respiratory Therapy, Byrdine F. Lewis College of Nursing and Health Professions, Georgia State University, Atlanta, GA USA; 3 Department of Health Promotion, College of Public Health, University of Kentucky, Lexington, KY USA; 4 Kinsey Institute for Research on Sex, Gender, and Reproduction, Indiana University, Bloomington, IN USA

**Keywords:** Transgender women, Suicidal ideation, Sexual abuse, Perceived stigma, Psychosocial impact of gender minority status

## Abstract

**Background:**

Transgender women (TGW) in the U.S. experience high rates of stigma, depression, and elevated rates of suicide. This study examined correlates of suicidal ideation and estimated the conditional indirect effects of perceived stigma and psychosocial mediators on suicidal ideation.

**Methods:**

Using a cross-sectional study design, TGW (*N* = 92) were recruited through snowball sampling in Atlanta, Georgia. Structured interviews were conducted. Suicidal ideation was assessed by combining two variables that measured suicidal thoughts. Logistic regression models were performed to identify the potential risk and protective factors for suicidal ideation. We examined hypothesized psychosocial factors, including anxiety, depression, psychosocial impact of gender minority status, and substance use behaviors as potential mediators for the relationship between perceived stigma and suicidal ideation. All models were controlled for age, race, education, and homelessness.

**Results:**

Suicidal ideation was reported by 33% (*N* = 30) of the study participants. In multivariable analysis, suicidal ideation was associated with sexual abuse (AOR = 3.17, 95% CI = 1.10–9.30), anxiety (AOR = 1.74, 95% CI = 1.10–2.73), family verbal abuse (AOR = 2.99, 95% CI = 1.10–8.40), stranger verbal abuse (AOR = 3.21, 95% CI = 1.02–10.08), and psychosocial impact of gender minority status (AOR = 3.42, 95% CI = 1.81–6.46). Partner support was found to be the protective factor for suicidal ideation (AOR = 0.34, 95% CI = 0.13–0.90)**.** In the mediation analysis, the psychosocial impact of gender minority status mediated the relationship between perceived stigma and suicidal ideation. The estimated conditional indirect effect was 0.46, (95% CI = 0.12–1.11).

**Conclusion:**

Interventions that aim to reduce suicidal behaviors among TGW should address stigma, psychosocial impact of gender minority status, and different forms of violence and abuse.

## Background

Transgender is an umbrella term for individuals whose gender identity or gender expression differs from what is typically associated with the sex that they were assigned at birth. The transgender community includes individuals, who were assigned male at birth and identify as female, who were assigned female at birth and identify as male, and who identify their gender as outside the binary categories of male or female [1–4]. In the United States, there are significant disparities in suicide risk based on gender identity. Transgender populations have elevated rates of suicidal ideation and suicide [5–7]. According to the US Trans Survey (USTS), attempted suicide was reported by 40% of transgender women (TGW) survey participants compared to 1.6% in the general population and 10.2% among Lesbian, Gay, and Bisexual (LGB) participants [5, 8]. Similarly, other studies found that 32.4 to 45.8% of transgender study participants reported lifetime suicide attempts [9, 10]. Suicidal ideation which is defined as “thinking about, considering, or planning for suicide” is also highly prevalent among TGW [11]. Multiple studies have found prevalence rates of suicidal ideation among TGW that range from 35.1 to 79.2% [9, 12, 13]. In one study, an alarming 78.1% of the participants reported suicidal ideation in the past year [14]. Adams et al. in 2017 conducted a meta-analysis and reported that, across the 23 studies that were conducted from 1997 to 2016, among the transgender population, the prevalence of lifetime suicidal ideation was reported by 55%, and suicidal ideation in the past 12 months was reported by 51% [15]. The same study also found higher lifetime suicidality among TGW (51.7%) compared to transgender men (45.4%), gender non-conforming individuals (30%), and cross-dressers (25.6%). As would be expected, suicidal ideation among TGW has been strongly associated with a history of suicide attempts [11, 16–18], and is a strong predictor of future suicide attempts [19]. Together, these findings highlight the need to identify the correlates of suicidal ideation among TGW, which would help to inform interventions to prevent suicidal ideation and suicide attempts.

Among cisgender populations, that is, people whose gender identity and gender expression align with their assigned sex at birth [20], research has identified predictors of suicidal ideation, including substance abuse, experiences of violence, depression, anxiety, and other mental health issues [18, 21–24]. For TGW, in addition to these factors, psychosocial factors that are specific to TGW, including stigma and discrimination, microaggressions, experiences of abuse and violence, family rejection, and lack of social support, could contribute to disproportionate rates of suicidality [12, 13, 25–27]. According to the USTS, transgender participants experienced various forms of discrimination due to their gender identity [5, 28], including unemployment (30%), being refused a home or apartment (23%), verbal harassment (46%), being denied equal treatment by a government agency or official (24%), and mistreatment by police (58%) [5, 8]. Several other studies have reported that TG individuals experience high levels of transgender-related discrimination and stigma [29]. For example, transgender individuals have elevated rates of being denied access to care, as well as experience verbal harassment and physical violence, when attempting to access doctors and hospitals, emergency rooms, and using ambulances/by Emergency Medical Technicians [5, 8].

The stigma and discrimination experienced due to their gender identity may be associated with several adverse health outcomes among TGW [30]. Stigma may be indirectly associated with poor mental health in TGW by restricting their access to healthcare, housing, and employment [31, 32]. There also is evidence of a direct association between stigma and stress and subsequent mental health problems [30, 33, 34]. Societal attitudes toward TGW and the discrimination experienced by TGW on a daily basis may have a significant psychosocial impact on TGW and could be an important factor in explaining the mechanism that leads to mental health issues and negative health behaviors. For example, there is evidence that perceived stigma and discrimination are associated with anxiety and depressive symptoms [35, 36], distress [33], suicide attempts [25], and a host of other negative mental outcomes [35, 37].

Minority stress theory states that sexual and gender minorities experience stressors, such as discrimination and stigma, that lead to increased levels of stress that can, in turn, deplete psychological resources (e.g., resilience, social support) and lead to poor overall mental and physical health outcomes [38]. In this theory, perceived stress is viewed as the mediator of the association between sexual/gender minority status and negative health outcomes. A complement to the minority stress theory is the psychological mediation framework, which postulates that sexual and gender minorities experience stigma-related stress that leads to certain intra-and interpersonal psychological processes that can affect mental health [39]. Within this framework, perceived stress relates to gender minority status and is the main predictor of negative health outcomes but with psychological and psychosocial factors that explain the association. Several studies have applied these theories to explain the disproportionate rates of mental health issues among sexual and gender minorities [9, 14, 40–42].

There is evidence that psychological and psychosocial factors, such as substance use, depression, and anxiety, may explain the association between perceived stigma and suicidal ideation [13]. Substance use has been hypothesized as a potential negative coping mechanism for the stress associated with stigma and discrimination among transgender persons. Substance use has demonstrated strong associations with suicidal ideation and suicide attempts [43] and has found to be a statistically significant mediator between stigma and suicidality among transgender individuals [43]. Moreover, the depression and anxiety associated with experiencing stigma and discrimination may lead to suicidal ideation and suicide attempts [8, 13]. Taken together, these psychosocial factors, including anxiety, depression, negative impact of gender minority status, and substance use behaviors could be hypothesized as mediators. In this study, our goal is to examine the relationship between perceived stigma by TGW, the psychosocial factors that include anxiety and depression, and the psychosocial impact of gender minority status, substance use behaviors, and suicidal ideation.

**Fig. 1:**
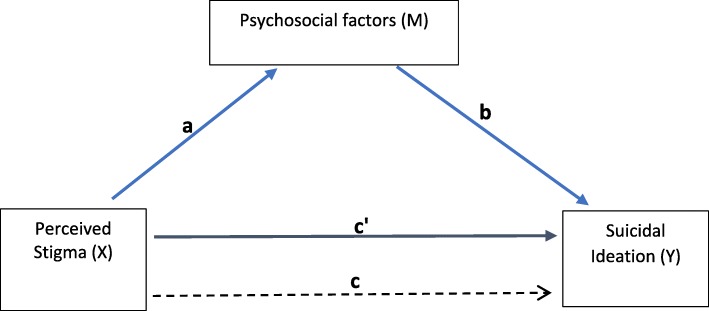
Conceptual Diagram of the mediation models. *Path c* - Total effect of perceived stigma (X) on suicidal ideation (Y). *path a -* Effects of perceived stigma (X) on psychosocial factors (Mediators (M): anxiety, depression, the psychosocial impact of gender minority status, excessive drinking, injection drug use, and non-injection drug use). *path b -* Effects of psychosocial factors (M) on suicidal ideation (Y) controlling for perceived stigma (X)*. path c’* - Direct effect of perceived stigma (X) on suicidal ideation (Y) controlling for psychosocial factors (M)

Evidence for the prevalence and correlates of suicidal ideation among TGW is found in the literature [12, 13, 25–27], but there is limited research [9, 14, 27, 41] on the role of psychosocial factors and the underlying mechanisms associated with suicidal ideation. This critical research gap needs to be addressed, as the findings could inform researchers and policymakers in designing suicide prevention interventions for TGW in the United States. In this study, we aim to measure the prevalence of suicidal ideation and to identify the demographic and psychosocial correlates of suicidal ideation and the potential underlying pathways associated with suicidal ideation among TGW. The conceptual framework (Fig. 1) shows our hypothesized model of the psychosocial factors that explain the pathway between perceived stigma and suicidal ideation. The psychosocial factors include anxiety, depression, the psychosocial impact of gender minority status, and substance use behaviors.

## Methods

### Participants and procedures

In this cross-sectional study, multiple community–based outreach strategies were used to recruit a sample of TGW (*n* = 92) between the ages of 18 and 65 years who reside in Atlanta, GA. Venues that serve TGW and word–of–mouth recommendations from transgender advocates provided the primary methods of recruitment. These venues offer HIV prevention and care, housing, and counseling services to the TGW. The study was known as the Transgender Atlanta Personal Survey. Transgender advocates notified the study project director when they located a woman who was willing to be screened for study participation. In addition, the project was advertised through formal and informal communication channels via advocacy groups and Lesbian, Gay, Bisexual, and Transgender (LGBT) service organizations. The project director used print materials to provide their contact information. Data were collected from August 2014 through June 2015.

TGW were screened to determine eligibility. The inclusion criteria were: (1) 18 to 65 years of age, (2) male sex assigned at birth, and (3) self–identifying as either female or transgender. All participants who were screened, except one individual who identified as “other” were eligible and consented to participate in the study. After providing written informed consent, women engaged in a face–to–face structured interview with a trained graduate research assistant. The training involved cultural-competency and the use of non-judgmental statements. Interview responses were recorded on a portable electronic tablet, using Qualtrics© software (Provo, Utah). The Institutional Review Board of Georgia State University approved study protocols following a full board review.

### Measures

The survey assessed sociodemographic characteristics, a broad range of theoretical contextual factors, and self-reported HIV status. In addtion, we assessed the prevalence of several trauma exposures, such as “ever experienced physical abuse by an intimate partner,” “ever being a victim of sexual abuse,” “ever experienced childhood sexual abuse,” and psychosocial factors.

Perceived stigma was assessed by using four items adapted for TGW from the original scale developed for gay individuals [44, 45]. These four items were a subscale that measured the TGW’s perceptions of society’s stigma or negative attitudes toward TGW. The items were: (1) “Society still punishes people for being transgender”; (2) “Most people have negative reactions to transgender people”; (3) “Discrimination against transgender people is still common”; and (4) “Only a few people discriminate against transgender people.” Response options were presented on a 5-point Likert scale, ranging from 1= strongly disagree to 5 =strongly agree. Item 4 was reverse coded. The mean of response scores for the four items were used for the analysis. Inter-item reliability was adequate (Cronbach’s alpha = 0.73).

The psychosocial impact of gender minority status was assessed using three items from a 4-item subscale developed by Sjoberg and colleagues [46]. The 4-item subscale is part of the longer Transgender Adaptation and Integration Measure and assessed four aspects of mental health related to transgender status. We used this subscale to measure psychosocial distress related to the unique experiences of TGW. The items were: (1) “I get depressed about my gender status”; (2) “My gender status interferes with my quality of life”; (3) “I have thought about suicide because of my gender status”; and (4) “Being transgender causes me relationship problems.” Response options were provided on a 5-point Likert scale, ranging from 1 = strongly disagree to 5 = strongly agree. Given that our outcome was suicidal ideation, the third item was not included in the analyses. We used the average of response scores for the three items. Inter-item reliability was adequate (Cronbach’s alpha = 0.71).

Suicidal ideation was assessed by combining two items that measured suicidal thoughts. The items were: (1) “In the past 12 months, have you considered attempting suicide?” for which the response options were Yes/No; and (2) “I have thought about suicide because of my gender status,” for which response options were provided on a 5-point Likert scale, ranging from 1 = strongly disagree to 5 = strongly agree. We dichotomized Item 2 by collapsing the responses of 4 (agree) and 5 (strongly agree) as “Yes” and all other responses as “No.” Then, we created a new variable, “suicidal ideation,” for the participants who responded “Yes” to either of the two items; these participants were considered as experiencing suicidal ideation and other participants, as not experiencing suicidal ideation.

Depression was measured using six items from the Brief Symptom Inventory [47]. This subscale is widely used as a psychological self-report symptom scale to measure depression. The items were: (1) “Feeling not interested in things”; (2) “Feeling lonely”; (3) “Feeling blue”; (4) “Feeling worthlessness”; (5) “Feeling hopeless about the future”; and (6) “Thoughts of ending your life.” Response options for all items were on a 5-point Likert scale, ranging from 1 = not at all to 5 = extremely. We calculated the mean of these six items as the depression score.

Anxiety was measured using the 3-item subscale from the Brief Symptom Inventory. The items were: (1) “Experienced nervousness or shakiness inside”; (2) “Feeling tense or keyed up”; and (3) “Feeling so restless you couldn’t sit still.” Response options for all items were on a 5-point Likert scale, ranging from 1 = not at all to 5 =extremely. For the anxiety score, we calculated the mean of the three items.

Excessive drinking was measured by three items: (1) “In the past 30 days, on how many days did you drink any alcohol?”; (2) “On the days when you drank alcohol in the past 30 days, about how many drinks did you have on average?”; and (3) “In the past 30 days, how many times did you have 5 or more alcoholic drinks in one sitting?” Based on the Dietary Guidelines for Americans, 2015–2020 [48], participants who consumed 15 or more drinks during the prior week or consumed more than 5 or more drinks in one sitting were considered to evidence excessive drinking.

Non-injection drug use was measured by one item: “In the past 12 months, have you used any non-injection drugs, other than those prescribed for you?” Response options were Yes/No.

Injection drug use was measured by one item: “Have you ever in your life shot up or injected any drugs other than those prescribed for you? By drugs, I am referring to drugs such as heroin, meth – not hormones or silicone? By shooting up, we mean anytime you might have used drugs with a needle, either by mainlining, skin popping, or muscling.” Response options were Yes/No.

Intimate partner violence is the experience of physical and emotional violence by a romantic or sexual partner in one's lifetime. This variable was measured by three items: (1) “In your lifetime, have you ever been physically abused by a romantic or sexual partner? By physical abuse, we mean a range of behaviors, from slapping, pushing, or shoving, to severe acts, such as being beaten, burned, or choked”; (2) “In your lifetime, have you ever been emotionally abused by a romantic or sexual partner? By l emotional abuse, we mean name-calling, or humiliating you, or trying to monitor and control or threaten you”; and (3) “Have you ever been physically abused or beaten by a romantic or sexual partner because of your gender identity or presentation?” Response options were Yes/No.

Sexual abuse is the experience of forced oral/anal sex in one's lifetime. This variable was measured by three items: (1) “In your lifetime, has someone ever made you perform oral sex?”; (2) “In your lifetime, has someone ever made you receive anal sex? By receiving anal sex, we mean they put their penis in your anus (you were the bottom)”; and (3) “In your lifetime, has someone ever made you perform anal sex? By performing anal sex, we mean they made you put your penis in their anus (you were the top)”. Response options were Yes/No.

Child sexual abuse was measured by one item: “As a child (less than 16 years old), were you ever sexually abused?” Response options were Yes/No/I do not remember. HIV status was measured by one item, “What was the result of your most recent HIV test?” for which the response options were “negative”/ “positive”/ “I do not know my status”.

### Data analysis

Descriptive statistics were computed among TGW who reported suicidal ideation and those who reported no suicidal ideation. To estimate bivariate associations, TGW with suicidal ideation were compared to TGW without suicidal ideation, using chi-square analyses and Fisher’s exact test for categorical variables (i.e., education, race, homelessness, excessive drinking, non-injection, injection drug use, and HIV status). Wilcoxon rank-sum tests were used for continuous variables (i.e., age, perceived stigma, anxiety, depression, and psychosocial impact of gender minority status). Control variables were selected based on the literature and the presence of statistically significant differences in our bivariate analyses (alpha = 0.10). Age, race, and education were statistically significantly different between both groups, and homelessness was associated with suicidal ideation among TGW in prior studies. To estimate the association of substance abuse behaviors, violence, abuse, HIV status, and other psychosocial factors with suicidal ideation, we conducted separate multivariable logistic regression models, adjusting for age, race, education, and homelessness.

We also examined the impact of perceived stigma on suicidal ideation through mediation analyses. A mediator is a variable that explains, or accounts for, the effect of the independent variable on the dependent variable [49, 50]. To examine the underlying mechanism between perceived stigma and suicidal ideation, we tested the role of six psychosocial factors as potential mediators to explain the effect of perceived stigma (Variable X) on suicidal ideation (Variable Y) (Fig. 1): anxiety, depression, the psychosocial impact of gender minority status, excessive drinking, injection drug use, and non-injection drug use (Variables M). Six separate mediation models were tested, one for each psychosocial factor. The effect of X on Y is the total effect (*path c*); the effect of X on M is indicated by *path a*; the effect of M on Y controlling for X, is indicated by *path b*; and the direct effect of X on Y, controlling for M, is *path c’*. The indirect effect is the product of *path a* and *path b*, which is *path ab*. The equation (*c = c’ + ab*), which indicates the total effect is equal to direct and indirect effects, does not hold true due to the use of logistic regression. By standardizing the coefficients expressed on a log-odds metric (multiplied by the standard deviation of the predictor variable and divided by standard deviation of outcome variable), however, *c* would be approximately equal to *c’ + ab* [49]. Using PROCESS macros v3.3 by Andrew F. Hayes [51], we tested the psychosocial impact of gender minority status, anxiety, and depression as mediators, and the point estimates for *path a, path b, path c’*, and *path c* were generated. For testing the dichotomous mediators (excessive drinking, injection drug use and non-injection drug use) we used the INDIRECT macro [50]. Bootstrapping (*N*= 5000) was used to construct confidence intervals (CIs) for the indirect effect (*path ab*) to determine statistically significant mediators. The Statistical Package for Social Sciences (SPSS), version 25.0, (IBM, Chicago, IL), was used for all analyses.

## Results

Among the total study sample of TGW (*N*= 92), the prevalence of suicidal ideation was 33% (*n*= 30). The average age of participants was 35 years, 51% were homeless, 60% of the participants who are aware of their HIV status reported as HIV positive, 50% had more than a high-school education, and 84% identified as Black or African American (Table 1). Of the study participants, sexual abuse was reported by 62%, and child sex abuse was reported by 52%. Substance abuse behaviors included excessive drinking (21%), injection drug use (15%), and non-injection drug use (34%). Among the total study sample, the mean score for the perceived stigma measure was 4.03 (SD = 0.66, range: 1–5), mean score of anxiety was 2.11 (SD = 1.14, range: 1–5), mean score of depression was 2.01 (SD = 0.85, range: 1–5), and mean score on the psychosocial impact of gender minority status measure was 2.63 (SD = 0.94, range: 1–5). Family verbal abuse and stranger verbal abuse were reported by 53% and 63% of the study participants, respectively. As shown in Table 1, significant differences between TGW who experiencd sucidial ideation and those who had not were found on a host of demographic and psychosocial variables.

**Table 1:** Descriptive statistics by suicidal ideation among transwomen residing in Atlanta, Georgia

Characteristic	Suicidal Ideation	Suicidal Ideation	Total
	(Yes), N = 30 (33%)	(No), *N* = 62 (67%)	*N* = 92 (%)
Age, Median (IQR)**	32 (11)	36 (11)	35 (11)
Education*			
High school or less	11 (37)	35 (57)	46 (50)
College or higher	19 (63)	27 (43)	46 (50)
Race**			
African American/ Black	18 (60)	59 (95)	77 (84)
Caucasian/ White	8 (27)	2 (3)	10 (11)
Others	4 (13)	1 (2)	5 (5)
Homeless^a^			
Yes	15 (50)	31 (51)	46 (51)
No	15 (50)	30 (49)	45 (49)
Non-injection drug use**^a^			
Yes	15 (52)	16 (26)	31 (34)
No	14 (48)	46 (74)	60 (66)
Injection drug use			
Yes	4 (13)	10 (16)	14 (15)
No	26 (87)	52 (84)	78 (85)
Excessive drinking			
Yes	9 (30)	10 (16)	19 (21)
No	21 (70)	52 (84)	73 (79)
Sexual abuse by any perpetrator (lifetime)**			
Yes	23 (77)	34 (55)	57 (62)
No	7 (23)	28 (45)	35 (38)
Intimate partner violence (lifetime)**			
Yes	28 (93)	45 (73)	73 (79)
No	2 (7)	17 (27)	19 (21)
Child Sex Abuse (less than 16 years old)*^a^			
Yes	19 (66)	28 (46)	47 (52)
No	10 (34)	33 (54)	43 (48)
Mean (Standard Deviation)			
Anxiety (3 items) (Range 1 to 5)**	2.54 (1.38)	1.90 (0.96)	2.11 (1.14)
Depression (6 items) (Range 1 to 5)**	2.24 (0.79)	1.90 (0.86)	2.01 (0.85)
Perceived stigma (4 items) (Range 1 to 5)**	4.23 (0.63)	3.92 (0.66)	4.03 (0.66)
Psychosocial impact of gender minority status (3 items) (Range 1 to 5) **	3.26 (0.97)	2.33 (0.77)	2.63 (0.94)
Family verbal abuse**			
Yes	21 (70)	28 (45)	49 (53)
No	9 (30)	34 (55)	43 (47)
Stranger verbal abuse**			
Yes	25 (83)	38 (61)	63 (68)
No	5 (17)	24 (39)	29 (32)
HIV**^,a^			
Positive	10 (40)	40 (60)	50 (60)
Negative	15 (60)	18 (31)	33 (40)
Partner Support**			
Yes	15 (50)	48 (77)	63 (68)
No	15 (50)	14 (23)	29 (32)

** *p* – value < 0.05, * *p* – value < 0.10, IQR – Inter Quartile Range, ^a^ Total may not add up due to missing data

In the multivariable analysis (Table 2), suicidal ideation was associated with sexual abuse (AOR: 3.17, 95% CI: 1.10, 9.30), higher anxiety scores (AOR: 1.74; 95% CI: 1.10, 2.73), psychosocial impact of gender minority status (AOR: 3.42, 95% CI: 1.81, 6.46), family verbal abuse (AOR: 2.99; 95% CI: 1.10, 8.40), stranger verbal abuse (AOR: 3.21; 95% CI: 1.02, 10.08), and partner support (AOR: 0.34; 95% CI: 0.13, 0.90). Depression, perceived stigma, non-injection drug use, intimate partner violence, child sex abuse, and HIV status were no longer associated with suicidal ideation in the multivariable analyses.

**Table 2:** Associations of suicidal ideation with risk factors among transwomen

Characteristic	Adjusted OR^a^ (95% CI)
Non-injection drug use	
Yes	2.30 (0.81–6.53)
No	1.00
Injection drug use	
Yes	1.28 (0.31–5.23)
No	1.00
Excessive drinking	
Yes	2.07 (0.58–7.35)
No	1.00
Sexual abuse	
Yes	3.17 (1.10–9.30) *
No	1.00
Intimate partner violence	
Yes	4.59 (0.89–23.66)
No	1.00
Child Sex Abuse	
Yes	1.91 (0.72–5.12)
No	1.00
Anxiety	1.74 (1.10–2.73) *
Depression	1.54 (0.83–2.80)
Perceived stigma	1.75 (0.81–3.76)
Psychosocial impact of gender minority status	3.42 (1.81–6.46)*
Family verbal abuse	
Yes	2.99 (1.10–8.40)*
No	1.00
Stranger verbal abuse	
Yes	3.21 (1.02–10.08)*
No	1.00
HIV status	
Positive	0.37 (0.13–1.10)
Negative	1.00
Partner Support	
Yes	0.34 (0.13–0.90)*
No	1.00

^a^Adjusted for age, race, education, and homelessness. *p – value < 0.05, OR – Odds Ratio, CI – Confidence Intervals

In the mediation analyses (Table 3), results showed that perceived stigma had a significant direct effect on the psychosocial impact of gender minority status (*path a*), (0.38, SE = 0.15, 95% CI = 0.08.0.68). The psychosocial impact of gender minority status also was significantly associated with suicidal ideation (*path b*) (1.19, SE = 0.33, 95% CI = 0.53,1.85), and there was a significant indirect effect (*path ab*) of the psychosocial impact of gender minority status on the association between perceived stigma and suicidal ideation (0.46, SE = 0.26, 95% CI: 0.12, 1.11). The other psychosocial mediator variables, anxiety, depression, excessive drinking, injection drug use, and non-injection drug use, were not significant mediators. The mediation effect size was estimated by calculating the ratio of (path ab/path c) [52]. The psychosocial impact of gender minority status mediated 77% of the effect of perceived stigma on suicidal ideation.

**Table 3:** Summary of mediation effects of psychosocial factors between perceived stigma and suicidal ideation

Mediator	*path a*, SE, 95% CI	*path b*, SE, 95% CI	*path c’*, SE, 95% CI	*path ab*, SE, 95% CI
Psychosocial impact of gender minority status	0.38*, 0.15, 0.08–0.68	1.19*, 0.33, 0.53–1.85	0.12, 0.45, −0.75 – 1.00.	0.46*, 0.26, 0.12–1.11
Anxiety	0.36, 0.18, − 0.005 – 0.72	0.48*, 0.23, 0.02–0.93	0.42, 0.41, − 0.38 – 1.23	0.17, 0.16, − 0.02 – 0.60
Depression	0.17, 0.14, − 0.11 – 0.45	0.40, 0.28, − 0.16 – 0.96	0.55, 0.40, − 0.24 – 1.34	0.06, 0.09, − 0.11 – 0.28
Excessive drinking	−0.04, 0.06, − 0.15 – 0.07	0.84, 0.60, − 0.33 – 2.01	0.62, 0.40, − 0.16 – 1.40	−0.03, 0.08, − 0.33 – 0.06
Injection drug use	− 0.02, 0.05, − 0.11 – 0.07	0.30, 0.72, −1.11 – 1.71	0.57, 0.39, − 0.19 – 1.33	−0.006, 0.18, − 0.25 – 0.09
Non-injection drug use	0.05, 0.07, − 0.09 – 0.19	− 1.01, 0.55, −2.09 – 0.07	0.81, 0.42, − 0.01 – 1.63	−0.05, 0.09, − 0.36 – 0.06

*Models were adjusted for age, homelessness, education, and race. All effects are on a log-odds metric,*

*p < 0.05, SE Standard Error, *CI* Confidence intervals

path *a* - Effects of perceived stigma on psychosocial factors,

path b - Effects of psychosocial factor on suicidal ideation,

path c’ - Direct effects of perceived stigma on suicidal ideation

path ab - Indirect effects of perceived stigma on suicidal ideation

## Discussion

In this study, we sought to determine the correlates for suicidal ideation among TGW and examined the mediation pathways that explain the underlying relationships. In our sample, the prevalence of suicidal ideation was 33%, within the range reported by other studies [13, 53]. We found that psychosocial factors, including anxiety, perceived stigma of being transgender, the psychosocial impact of gender minority status, experiencing sexual abuse, family verbal abuse, and stranger verbal abuse were significantly associated with higher odds of suicidal ideation. Partner support was found to be a protective factor.

Using the postulates of minority stress theory and the psychological mediation framework, we investigated whether certain psychosocial factors explained the associations between perceived stigma and suicidal ideation. We found that the psychosocial impact of gender minority status was a statistically significant mediator in our sample, indicating that the effect of perceived stigma on suicidal ideation may be explained by this pathway. The other psychosocial mediators examined, depression, anxiety, and substance use behaviors, were not statistically significant, which is contrary to the findings of previous research [43].

The risk factors that we found to be significantly associated with suicidal ideation are in keeping with those of other studies that reported a lack of or low social support [7, 13, 54], sexual abuse and gender-based discrimination [25], family verbal abuse [12], stigma and discrimination [26], and the psychosocial impact of gender minority status and internalized transphobia [10]. Taken together, these societal-level risk factors suggest that TGW are at risk for a multitude of traumatic experiences that have severe mental health sequelae. Until there is a shift in societal attitudes and norms, TGW could benefit from public health interventions, such as mind-body programs [55] that enhance resiliency and improve coping, or online-eHealth interventions [56] that provide a safe space to improve skills and receive support. At a minimum, mental health professionals and social service providers who work with this population should be sensitive to the abuse history and mental health needs of the TGW with whom they work.

As would be expected, interpersonal factors, such as experiencing sexual abuse; psychosocial factors, such as anxiety and depression; and trans-specific factors, such as perceived stigma, family and stranger verbal abuse due to gender identity; and the psychosocial impact of gender minority status predicted the likelihood of suicidal ideation among TGW. We also found that partner support was a protective factor among TGW, similar to other studies that found a reduced risk of suicidal ideation among the TGW with higher levels of social support [13], indicating the vital role of a support system for TGW. These findings suggest that interpersonal, trans-specific, and psychosocial factors should be the focus in the development of suicide prevention interventions.

We found that the construct, the psychosocial impact of gender minority status, which measures the psychosocial distress experienced by TGW related to their unique gender identity, was significantly associated with suicidal ideation. The psychosocial impact of gender minority status is different from the other general forms of distress, such as anxiety, psychological distress, and depression [46], and represents the psychological impact of stigma and discrimination based on gender identity. This result indicates that this specific form of distress related to TGW is a significant predictor of suicidal ideation. Structural interventions to change the policies and laws to combat the stigma and discrimination against TGW are necessary.

We also found that the psychosocial impact of gender minority status was a statistically significant mediator that partially explains the relationship between perceived stigma and suicidal ideation. This finding was similar to the studies that: (a) reported that internalized trans-negativity (sometimes referred to as internalized transphobia) mediated the relationship between distal stressors (such as anti-trans stigma, discrimination and victimization) and suicidal ideation [40], and (b) applied the minority stress theory to TGW and found that, internal stressors (internalized transphobia) mediated the relationship between external distal stressors (anti-trans discrimination, stigma, and victimization) and suicidal ideation [9]. The model used in our study, however, was conceptualized differently from the frameworks applied in these previous studies. In our model, perceived stigma tapped into TGW’s *perception*s of the prejudice and discrimination in their community. In this context, perceived stigma could be viewed as a proxy for distal external and objective stressors. We found that perceived stigma nonetheless had an impact on psychological processes among the TGW in our study. The implication of this conceptualization is that perceptions of the societal anti-transgender attitudes and structural level anti-transgender polices are important to assess even if they are considered as proxies for distal objective measures of stressors and that, for some TGW, perceptions are important to their mental health. TGW who perceive transgender-related stigma in our study, experienced increased psychosocial effects that could eventually be manifested in behaviors such as suicidal ideation.

### Limitations

Although this study fills an important gap in the literature, several limitations exist. First, our study is a cross-sectional study, and we cannot infer causation between predictor variables and suicidal ideation. In particular, the lack of temporality limits the interpretation of the findings from the mediation model. Future studies should investigate these associations among TGW in a longitudinal framework. In addition, convenience sampling was utilized to recruit participants, and most of the participants were referred through the community-based organization that provides support services to TGW. Therefore, our sample may not be representative of TGW who live in Atlanta. Due to the small sample size, this study may not have adequate statistical power to detect some significant associations. Finally, although culturally competent interviewers were utilized, social desirability bias may have affected some of the sensitive responses from participants. Recall bias also may have affected the participants’ responses, particularly with regard to questions about early childhood.

## Conclusions

TGW are disproportionately affected by suicidal ideation and are in urgent need of tailored and effective interventions to ameliorate their mental health concerns. Interventions that increase social inclusion may be particularly beneficial [13]. In one intervention utilizing mHealth to promote social support among TGW, a significant decrease in depressive symptoms and an increase in social inclusion was observed [56]. Moreover, policy-level interventions that aim at decreasing stigma, discrimination, and transphobia are likely to positively impact transgender individuals [13]. Education on gender diversity may also mitigate transphobia and raise awareness on gender identity [40]. Specifically, clinicians and psychologists should be culturally competent in the psychological issues and gender identity specific to TGW [41]. Clinicians should also use Minority Stress Theory as a framework for assessing and treating their gender minority patients. They also need to consider how experiences and perceptions of stigma and discrimination are chronic stressors that TGW must cope with and ultimately may manifest in internalized processes that contribute to risk behaviors and negative mental health outcomes [41, 57]. Further, clinicians should support resiliency factors that are incorporated in minority stress theory, including group social support and encourage connecting to sexual and gender minority communities. Individual-level change also is critical to address the internalized stigma and trans-negativity among TGW. Based on the high levels of internalized stigma and the strong association with negative mental health outcomes, mental health professionals should work with TGW to address trans-negativity and provide healthy coping skills [40]. It is clear that structural-level interventions that seek to reduce stigma and discrimination and may have an effect on TGW’s perceptions and experiences are greatly needed. By changing societal attitudes, including those of healthcare providers and structures (e.g., clinics, systems, etc.), ultimately, negative psychosocial impacts will be lessened, and TGW will expereience improved access, engagement, quality of care, and mental health outcomes (including decreased risk of suicide). Future research should focus on creating and evaluating multilevel interventions to help reduce suicidal ideation among TGW as well as evaluating the effectiveness of treatment that is based on the minority stress framework to clinical practice.

## Data Availability

The datasets analyzed during the current study are not publicly available as it is a small pilot study and contain information that could compromise research participant privacy but are available from the corresponding author on reasonable request.

## Abbreviations

AOR: Adjusted Odds Ratio
CBO: Community Based Organization
CI: Confidence Intervals
LGBT: Lesbian Gay Bisexual, and Transgender
MSM: Men who have sex with men
OR: Odds Ratio
TGW: Transgender women
